# Radiomics Outperforms Conventional Tumor Volume in Prognostic Stratification Across Independent Glioma Cohorts

**DOI:** 10.7759/cureus.112298

**Published:** 2026-07-08

**Authors:** Umar Hashim, Abdullah Jalal, Ameer N Jabali, Hunaid Rana

**Affiliations:** 1 Chicago College of Osteopathic Medicine, Midwestern University, Hoffman Estates, USA; 2 Department of Foundational Medical Studies, Oakland University William Beaumont School of Medicine, Rochester, USA; 3 Department of Oncology, Midwestern University Chicago College of Osteopathic Medicine, Downers Grove, USA; 4 Department of Radiology, University of South Alabama, Mobile, USA

**Keywords:** external validation, glioblastoma, glioma, mri, radiomics, risk stratification, survival, tumor volume

## Abstract

Background: Tumor volume is routinely assessed on MRI and is often used as a surrogate measure of glioma burden. However, conventional volume measurements may not capture the spatial heterogeneity and phenotypic complexity visible on routine MRI. To address this potential gap in clinical assessment, we compared the prognostic performance of segmentation-derived tumor volume and that of an MRI-derived radiomic risk score across independent glioma cohorts. Materials and methods: This retrospective multi-cohort study included four publicly available glioma MRI cohorts comprising 1,447 patients: MU-Glioma-Post (n = 194), University of California San Diego post-treatment glioblastoma (UCSD-PTGBM; n = 178), University of California San Francisco preoperative diffuse glioma MRI (UCSF-PDGM; n = 494), and the University of Pennsylvania glioblastoma (UPenn-GBM; n = 581). MU-Glioma-Post served as the discovery cohort for post-treatment volumetric progression-free survival (PFS) analysis. UCSD-PTGBM, UCSF-PDGM, and UPenn-GBM were used for external volume assessment, radiomic validation, and head-to-head radiomics-versus-volume testing. Results: In the MU discovery cohort, 194 patients were included, and 152 experienced progression. Median PFS/follow-up was 159 days (IQR 77-283). Active tumor volume was independently associated with shorter PFS after adjustment for age, grade, isocitrate dehydrogenase status, and O6-methylguanine-DNA methyltransferase (MGMT) methylation (HR 1.045 per 10,000 mm³, 95% CI 1.015-1.076, p = 0.003; C-index 0.676). In external volume analyses, UCSD enhancing volume was not significantly associated with PFS (HR 1.144, 95% CI 0.935-1.398, p = 0.191), while UCSF fluid-attenuated inversion recovery volume was significant on univariable analysis (HR 0.917, 95% CI 0.875-0.961, p = 0.00031) but not after clinical adjustment (HR 1.034, 95% CI 0.983-1.087, p = 0.196). In UPenn-GBM, automated tumor volume was not associated with overall survival (OS) on univariable analysis (HR 0.993, 95% CI 0.978-1.009, p = 0.409) or adjusted analysis (HR 0.998, 95% CI 0.982-1.014, p = 0.768). In contrast, the radiomic score was associated with worse OS in UPenn (HR 1.634, 95% CI 1.302-2.050, p < 0.001). In direct head-to-head modeling, the radiomic score remained significant while tumor volume did not, both unadjusted (radiomics HR 1.626, p < 0.001; volume HR 0.996, p = 0.655) and adjusted for age, isocitrate dehydrogenase 1, and MGMT (radiomics HR 1.580, 95% CI 1.246-2.005, p < 0.001; volume HR 0.998, p = 0.806).

Conclusions: Conventional tumor volume showed inconsistent prognostic performance across external glioma cohorts and failed to retain independent significance in UPenn head-to-head models. MRI radiomics demonstrated greater prognostic reproducibility and remained independently associated with OS beyond that explained by tumor volume. These findings suggest that radiomic phenotyping captures clinically relevant imaging information beyond simple tumor burden measurements.

## Introduction

Gliomas remain among the most challenging primary central nervous system tumors due to their infiltrative growth, molecular heterogeneity, and variable clinical trajectories. Current prognostication relies on integrated histopathologic and molecular classification, including World Health Organization (WHO) grade, isocitrate dehydrogenase (IDH) status, O6-methylguanine-DNA methyltransferase (MGMT) promoter methylation, patient age, performance status, and extent of resection [1,2]. MRI remains central to diagnosis, treatment planning, response assessment, and longitudinal surveillance, particularly in the context of standard combined-modality therapy for glioblastoma [2,3]. However, conventional imaging interpretation may not fully capture the quantitative tumor phenotype that underlies clinical behavior.

Tumor volume is one of the simplest and most intuitive quantitative MRI biomarkers. Larger enhancing or nonenhancing tumor burden may reflect more extensive disease and is often assumed to carry prognostic significance. In clinical practice and research, volumetric measurements are attractive because they are interpretable, easy to communicate, and compatible with segmentation workflows already used for radiotherapy planning and treatment response assessment [2,3]. However, volume is a relatively coarse biomarker. Two tumors with similar volumes may differ substantially in shape, boundary complexity, necrosis, edema, texture, and spatial heterogeneity.

Radiomics provides a complementary quantitative imaging framework by extracting high-throughput features that describe tumor shape, intensity distribution, and texture [4-6]. In glioma, radiomic features have been investigated for molecular subtype prediction, grading, treatment response assessment, and survival stratification [7,8]. A persistent limitation is that many radiomic models are developed and tested on a single dataset, raising concerns about overfitting and limited clinical generalizability [9-12]. A clinically useful imaging biomarker should retain prognostic value across independent cohorts and demonstrate added information beyond simpler measurements such as tumor volume.

The purpose of this study was to compare conventional segmentation-derived tumor volume with an MRI-derived radiomic risk score across independent glioma cohorts. We hypothesized that tumor volume would show variable prognostic performance across datasets. In contrast, radiomic phenotyping would provide more reproducible prognostic information and remain associated with survival when directly modeled alongside tumor volume.

This study aims to (1) test whether post-treatment active tumor volume is independently associated with progression-free survival (PFS) in the MU-Glioma-Post discovery cohort; (2) assess whether conventional segmentation-derived tumor volume retains independent prognostic value across external glioma cohorts (University of California San Diego post-treatment glioblastoma (UCSD-PTGBM), University of California San Francisco preoperative diffuse glioma MRI (UCSF-PDGM), and University of Pennsylvania glioblastoma (UPenn-GBM)); and (3) determine, in a direct head-to-head model in UPenn-GBM, whether a locked MRI radiomic score provides prognostic information beyond automated tumor volume.

## Materials and methods

Study design and data sources

This retrospective multi-cohort imaging biomarker study used publicly available, de-identified glioma datasets. Cohorts were selected based on the availability of MRI segmentation masks, survival outcome data PFS or overall survival (OS)), and key clinical covariates (age, tumor grade, IDH status, and MGMT methylation status). Patients were included if they had a confirmed glioma diagnosis, available pre- or post-treatment MRI with segmentation masks, and complete survival outcome data. Patients were excluded if segmentation data were missing, clinical covariate data were incomplete for the planned adjusted models, or survival time could not be determined. Specifically, patients were dropped from a given cohort's analytic file if any of the following applied: no evaluable segmentation mask was available at the relevant timepoint (baseline for MU-Glioma-Post and UPenn-GBM or the designated imaging timepoint for UCSD-PTGBM); more than one of the four covariates (age, grade, IDH status, or MGMT methylation status) required for the adjusted model was missing; or PFS/OS time or event status could not be determined from the source clinical file. No additional volumetric or intensity-based quality-control thresholds beyond mask presence and plausibility (nonzero, anatomically bounded tumor volume) were applied.

Applying these criteria to the originally available cohort files (MU-Glioma-Post n = 194 screened/194 analyzed; UCSD-PTGBM n = 178 screened/178 analyzed; UCSF-PDGM n = 494 screened/494 analyzed; UPenn-GBM n = 581 screened/581 analyzed with successful automated radiomic score calculation) resulted in no additional exclusions beyond the source datasets' own inclusion criteria [13-16]. The MU-Glioma-Post cohort [13] was used as the post-treatment discovery cohort for volumetric PFS analysis. UCSD-PTGBM [14] and UCSF-PDGM were used for external volume analyses, and UPenn-GBM was used for external radiomic validation and direct head-to-head comparison between radiomics and conventional tumor volume. UCSF-PDGM and UPenn-GBM have been previously described as publicly available glioma MRI resources [15,16]. Because all datasets were publicly available and de-identified, no direct patient contact or new recruitment was performed.

**Figure 1:**
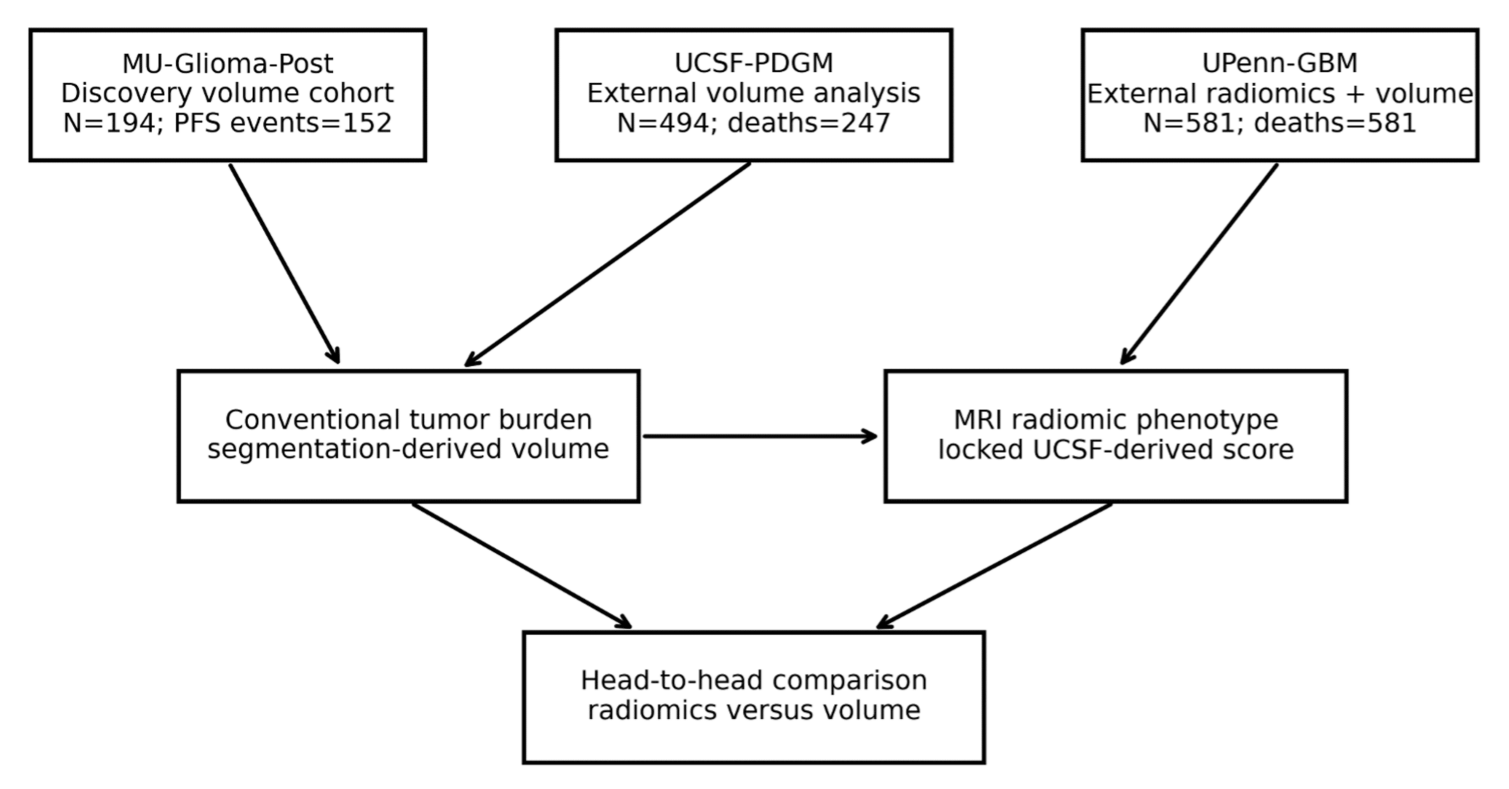
Study workflow showing the overview of cohort selection, discovery volume analysis, external volume analyses, radiomic score validation, and UPenn head-to-head radiomics-versus-volume testing UCSD-PTGBM: University of California San Diego post-treatment glioblastoma, UCSF-PDGM: University of California San Francisco preoperative diffuse glioma magnetic resonance imaging, UPenn-GBM: University of Pennsylvania glioblastoma

The primary scientific question was whether radiomics provides prognostic information beyond simple tumor volume. Accordingly, the study was organized into three analytic components: (1) discovery testing of post-treatment active tumor volume in MU-Glioma-Post, (2) external assessment of volume across UCSD-PTGBM, UCSF-PDGM, and UPenn-GBM, and (3) head-to-head comparison of a locked radiomic score versus automated tumor volume in UPenn-GBM (Figure 1).

Cohorts and endpoints

In the MU-Glioma-Post discovery cohort, the primary endpoint was PFS. The MU-Glioma-Post dataset was retrospectively assembled from pathologically confirmed glioma cases collected over approximately 10 years at the University of Missouri [13]. Follow-up time was defined as the time to first progression for patients who progressed and as the last MRI follow-up day for non-progressors. The final analytic cohort included 194 patients with complete PFS, event, covariate, and active tumor volume data. Progression occurred in 152 patients; 42 patients were censored. Median follow-up/PFS time was 159 days (interquartile range (IQR) 77-283).

In UCSD-PTGBM, the endpoint was PFS. The UCSD-PTGBM dataset included 178 subjects and 243 imaging time points as previously described [14]. In UCSF-PDGM, the endpoint was OS, with 247 deaths among 494 patients. Median OS was 421 days (IQR 187-754). Patient accrual for UCSF-PDGM occurred between 2015 and 2021 [15]. UCSF volume analyses used T1 contrast-enhanced (T1c) and fluid-attenuated inversion recovery (FLAIR) segmentation-derived mesh/voxel volumes [15]. In UPenn-GBM, OS was evaluated in 581 patients with successful automated radiomic score calculation and matched clinical information. Median OS was 392 days (IQR 175-642). Patient accrual for UPenn-GBM occurred between 2006 and 2018 [16]. Importantly, the UPenn validation file as structured contains observed death events for all 581 included patients (100% event rate), indicating that the available dataset reflects an ascertainment-censored structure in which only patients with confirmed death outcomes were retained. This configuration precludes true censoring and may introduce selection bias toward shorter-surviving patients. This limitation is explicitly considered in the interpretation of UPenn-GBM results, and findings from this cohort should be understood as internally consistent prognostic associations rather than estimates generalizable to a prospective or consecutive patient population [16].

Tumor volume extraction

For the MU discovery cohort, baseline post-treatment segmentation volumes were used to derive active tumor volume. Segmentations for MU-Glioma-Post were derived using the dataset's published automated multi-sequence segmentation pipeline (deep-learning-based, multi-label enhancing/non-enhancing/edema masks) rather than manual delineation, as detailed in the original dataset publication [13]; active tumor volume in the present analysis corresponds to the enhancing-plus-non-enhancing ("active") label from that automated segmentation. Underlying MRI acquisition followed standard clinical post-treatment glioma surveillance protocols (T1, post-contrast T1, T2, and FLAIR sequences on clinical 1.5T/3T scanners); scanner manufacturer, field strength, and full sequence parameters varied across patients and time as part of routine multi-year clinical care and are reported in detail in the original dataset publication [13] rather than reproduced here. Active tumor volume was scaled per 10,000 mm³ to improve interpretability of Cox coefficients. In UCSD-PTGBM, tumor volume was extracted from the available segmentation masks and scaled to 10,000 mm³. In UCSF-PDGM, T1c and FLAIR mesh and voxel volume variables were available from radiomic feature-extraction outputs and were similarly scaled to 10,000 mm³ [15]. For UPenn-GBM, automated segmentation masks were used to calculate tumor volume directly from voxel counts multiplied by voxel dimensions, and volumes were merged to the external validation dataset by patient identifier [16].

Volume models were intentionally kept simple to reflect conventional tumor burden measurement rather than high-dimensional imaging phenotyping. The primary volume terms were active tumor volume for MU-Glioma-Post, enhancing volume for UCSD-PTGBM, FLAIR/T1c volume for UCSF-PDGM, and automated tumor volume for UPenn-GBM.

Radiomic score and validation

The MRI radiomic risk score was derived from a locked UCSF-based formula developed by the present study’s authors in a prior internal analysis using the UCSF-PDGM dataset; that development analysis has not been previously published as a separate manuscript and is described here for completeness. In that development analysis, radiomic features were extracted from T1c and FLAIR sequences using PyRadiomics version 3.1 (Dana-Farber Cancer Institute, Brigham and Women's Hospital, Harvard Medical School, Boston, MA, USA), following Image Biomarker Standardization Initiative-concordant defaults [9] with the following fixed extraction settings applied uniformly to all images: images and masks were resampled to an isotropic voxel size of 1 × 1 × 1 mm³ using B-spline interpolation for the image and nearest-neighbor interpolation for the mask; intensities were discretized using a fixed bin width of 25 (no separate z-score normalization was applied beyond standard clinical intensity scaling); only original (non-filtered) image features were used, that is, no wavelet, Laplacian-of-Gaussian, or other derived-image filters were included; and shape, first-order, gray-level co-occurrence matrix (GLCM), and gray-level size-zone matrix (GLSZM) feature classes were computed using PyRadiomics default distance and symmetry settings. The complete extraction configuration file (PyRadiomics params.yaml) is available from the corresponding author upon request and will be deposited alongside the analysis code. A Cox proportional hazards model with least absolute shrinkage and selection operator-penalized feature selection was then used to identify prognostic features and estimate coefficients for OS. The resulting six-feature linear score was locked, with coefficients fixed at their UCSF-derived values, prior to any application to external data. The score incorporated T1c shape features (flatness, sphericity), T1c texture features (GLCM difference entropy, GLCM MCC, GLSZM small-area emphasis), and a FLAIR gray-level size-zone feature (size-zone nonuniformity). The formula was applied directly to UPenn-GBM without additional feature selection, coefficient refitting, or retraining. This locked-score design was used to reduce overfitting and evaluate transportability across institutions, consistent with reproducibility concerns emphasized in radiomics and prediction-model reporting literature [9-12]. The individual feature terms and locked coefficients are listed in Table 1. The locked radiomic score was calculated as radiomic score = 1.112652 − 0.617126(T1c_flatness) − 1.748229(T1c_sphericity) + 0.332631(T1c_GLCM_difference_entropy) − 1.340750(T1c_GLCM_MCC) − 0.284865(T1c_GLSZM_small_area_emphasis) − 0.000054(FLAIR_GLSZM_size_zone_nonuniformity).

**Table 1:** Locked radiomic score formula terms and available coefficients used for external validation Locked radiomic score formula terms and coefficients applied to the UPenn-GBM external validation cohort without feature reselection, coefficient refitting, or model retraining. UPenn-GBM: University of Pennsylvania glioblastoma

Radiomic score term	Coefficient
(Intercept)	1.112652
T1c_original_shape_Flatness	-0.617126
T1c_original_shape_Sphericity	-1.748229
T1c_original_glcm_DifferenceEntropy	0.332631
T1c_original_glcm_MCC	-1.340750
T1c_original_glszm_SmallAreaEmphasis	-0.284865
FLAIR_original_shape_Flatness	Not included in final locked formula
FLAIR_original_glszm_SizeZoneNonUniformity	-0.000054

Primary UPenn radiomic validation used automated segmentations. Manual-segmentation sensitivity analysis was performed in the subset of patients with successful manual radiomic score extraction. Automated and manual segmentation-derived radiomic scores were compared among overlapping patients using Pearson and Spearman correlation.

Statistical analysis

All statistical analyses were performed using R version 4.6.0 (R Foundation for Statistical Computing, Vienna, Austria) and RStudio version 2026.05.1+225 (Posit Software, PBC, Boston, MA, USA), including the survival package. Model discrimination was summarized using the concordance index (C-index), a measure of discrimination ranging from 0.5 (no discrimination) to 1.0 (perfect discrimination). Model fit was compared using the partial Akaike information criterion (AIC) when available. Bootstrap resampling with 1,000 successful iterations was used to assess the stability of UPenn radiomic associations and clinical/radiomic model comparisons. Kaplan-Meier (KM) curves were generated using median-based risk stratification for visual display. Statistical significance was interpreted as p < 0.05 (two-sided). Reporting of model performance and validation was guided by established prediction-model reporting principles [10,11].

## Results

Cohort characteristics

The MU discovery cohort contained 194 patients in the final PFS model, with 152 progression events and 42 censored observations. The cohort was predominantly high grade, with 160 grade four tumors (82.5%). UCSF-PDGM included 494 patients, 247 deaths, and balanced censoring/death status. UPenn-GBM included 581 patients in the automated external radiomic validation and volume comparison dataset. Baseline cohort characteristics are summarized in Table 2.

**Table 2:** Baseline and analytic characteristics of the three principal cohorts Baseline and analytic characteristics of the discovery and external validation cohorts. MU-Glioma-Post coding reflects the cleaned analysis file, where binary variables were coded as 0 = absence of the characteristic (e.g., IDH wildtype, MGMT unmethylated); 1 = presence of the characteristic (e.g., IDH mutated, MGMT methylated); and 2 = a second mutated/altered category for IDH (e.g., IDH-altered not otherwise specified). The UPenn-GBM validation file contained observed death events for all included patients, which is addressed as a limitation. IQR: interquartile range, SD: standard deviation, WHO: World Health Organization, CNS: central nervous system, GTR: gross total resection, STR: subtotal resection, NOS: not otherwise specified, NEC: not elsewhere classified, IDH: isocitrate dehydrogenase, MGMT: O6-methylguanine-DNA methyltransferase, UPenn-GBM: University of Pennsylvania glioblastoma, UCSF-PDGM: University of California San Francisco preoperative diffuse Glioma magnetic resonance imaging, OS: overall survival, PFS: progression-free survival

Characteristic	MU-Glioma-Post discovery	UCSF-PDGM	UPenn-GBM
Analytic N	194	494	581
Endpoint	PFS	OS	OS
Events	152 progression events (78.4%)	247 deaths (50.0%)	581 deaths (100.0%)
Median outcome time, days (IQR)	159 (77-283)	421 (187-754)	392 (175-642)
Age, years, mean ± SD	57.3 ± 16.0	56.8 ± 15.1	63.4 ± 11.9
Male sex	Not available in cleaned file	295 (59.7%)	Not available in validation file
WHO/CNS grade 4	160 (82.5%)	395 (80.0%)	GBM cohort
IDH wildtype/non-mutated	153 coded 0 (78.9%)	391 wildtype (79.1%)	478 wildtype (82.3%)
IDH mutated/altered	41 coded 1/2 (21.1%)	103 non-wildtype (20.9%)	10 mutated; 93 NOS/NEC
MGMT methylated/positive	63 coded 1 (32.5%)	296 positive (59.9%)	102 methylated (17.6%)
MGMT unmethylated/negative	92 coded 0 (47.4%)	113 negative (22.9%)	149 unmethylated (25.6%)
Extent of resection	Not available in cleaned file	GTR 244; STR 196; biopsy 54	GTR >90%: 345 yes; 202 no

Discovery cohort: active tumor volume PFS

**Figure 2:**
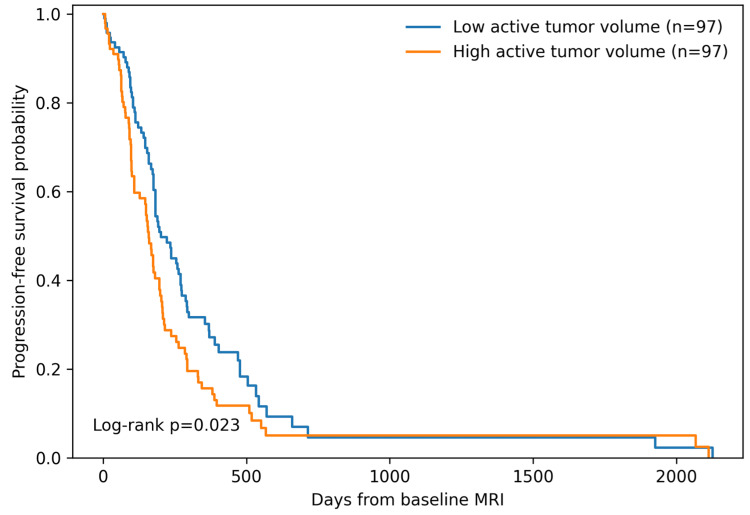
KM curve for MU-Glioma-Post discovery cohort stratified by median active tumor volume Higher active tumor volume was associated with worse PFS (log-rank p = 0.023). KM: Kaplan-Meier, MRI: magnetic resonance imaging, PFS: progression-free survival

In the MU post-treatment discovery cohort, active tumor volume was independently associated with shorter PFS. In the adjusted Cox model including age at diagnosis, grade, IDH status, and MGMT promoter methylation, each 10,000 mm³ increase in active tumor volume was associated with a 4.5% increase in the hazard of progression (HR 1.045; 95% CI, 1.015-1.076; p = 0.003). The adjusted model C-index was 0.676. MGMT promoter methylation was also associated with lower progression hazard (HR 0.855; 95% CI, 0.741-0.987; p = 0.033). Median-based KM analysis demonstrated a significant difference between the high- and low-active tumor volume groups (log-rank p = 0.023; Figure 2).

External volume analyses showed inconsistent prognostic performance

**Figure 3:**
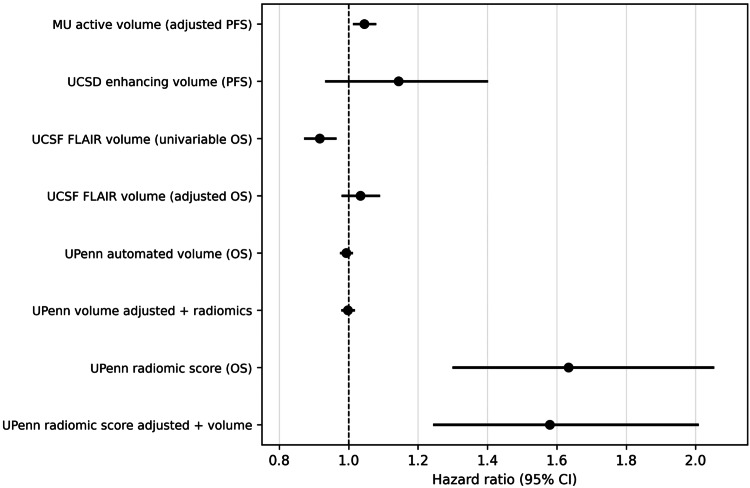
Forest plot summarizing volume and radiomic HRs across cohorts HRs and 95% CIs were estimated using Cox proportional hazards regression. Volume effects were inconsistent, while UPenn radiomics remained significant in univariable and adjusted head-to-head analyses. HRs: hazard ratios, CIs: confidence intervals, UCSD: University of California San Diego, UCSF: University of California San Francisco, UPenn: University of Pennsylvania, FLAIR: fluid-attenuated inversion recovery, PFS: progression-free survival, OS: overall survival, CI: confidence interval

The prognostic value of tumor volume did not generalize consistently across external cohorts (Table 3; Figure 3). In UCSD-PTGBM, enhancing tumor volume was not significantly associated with PFS (HR 1.144; 95% CI, 0.935-1.398; p = 0.191; C-index 0.556). In UCSF-PDGM, FLAIR and T1c volume showed a statistically significant univariable association with OS (FLAIR mesh volume HR 0.917; 95% CI, 0.875-0.961; p = 0.00031; C-index 0.565), but the volume association was attenuated and no longer significant after adjustment for age, WHO CNS grade, IDH status, and MGMT methylation status (FLAIR mesh volume adjusted HR 1.034; 95% CI, 0.983-1.087; p = 0.196). In UPenn-GBM, automated tumor volume was not associated with OS in univariable analysis (HR 0.993; 95% CI, 0.978-1.009; p = 0.409; C-index 0.504) or in adjusted analysis (HR 0.998; 95% CI, 0.982-1.014; p = 0.768).

**Table 3:** Prognostic performance of conventional tumor volume across cohorts HRs and 95% CIs were estimated using Cox proportional hazards regression. Multivariable (adjusted) models included the following covariates: MU-Glioma-Post, adjusted for age at diagnosis, tumor grade, IDH status, and MGMT promoter methylation; and UCSF-PDGM, adjusted for age at MRI, WHO CNS grade, IDH status, and MGMT methylation status. C-index (concordance index) is a measure of model discrimination ranging from 0.5 (no discrimination) to 1.0 (perfect discrimination); values labeled “model” refer to the discrimination of the full multivariable model including all covariates (not the biomarker alone). Conventional tumor volume showed prognostic value in the MU discovery cohort and in the UCSF univariable analysis but did not consistently retain independent prognostic value across external validation settings. PFS: progression-free survival, OS: overall survival, FLAIR: fluid-attenuated inversion recovery, T1c: T1 contrast-enhanced, UCSD-PTGBM: University of California San Diego post-treatment glioblastoma, UPenn-GBM: University of Pennsylvania glioblastoma, HRs: hazard ratios, CIs: confidence intervals, IDH: isocitrate dehydrogenase, MGMT: O6-methylguanine-DNA methyltransferase, WHO: World Health Organization, CNS: central nervous system

Cohort/model	Endpoint	Biomarker	HR (95% CI)	p-value	C-index
MU adjusted discovery	PFS	Active tumor volume per 10,000 mm³	1.045 (1.015-1.076)	0.003	0.676
UCSD univariable	PFS	Enhancing volume per 10,000 mm³	1.144 (0.935-1.398)	0.191	0.556
UCSF univariable	OS	FLAIR mesh volume per 10,000 mm³	0.917 (0.875-0.961)	0.00031	0.565
UCSF adjusted	OS	FLAIR mesh volume per 10,000 mm³	1.034 (0.983-1.087)	0.196	0.722 model
UPenn univariable	OS	Automated tumor volume per 10,000 mm³	0.993 (0.978-1.009)	0.409	0.504
UPenn adjusted	OS	Automated tumor volume per 10,000 mm³	0.998 (0.982-1.014)	0.768	0.645 model

Radiomic score externally validated in UPenn-GBM

**Figure 4:**
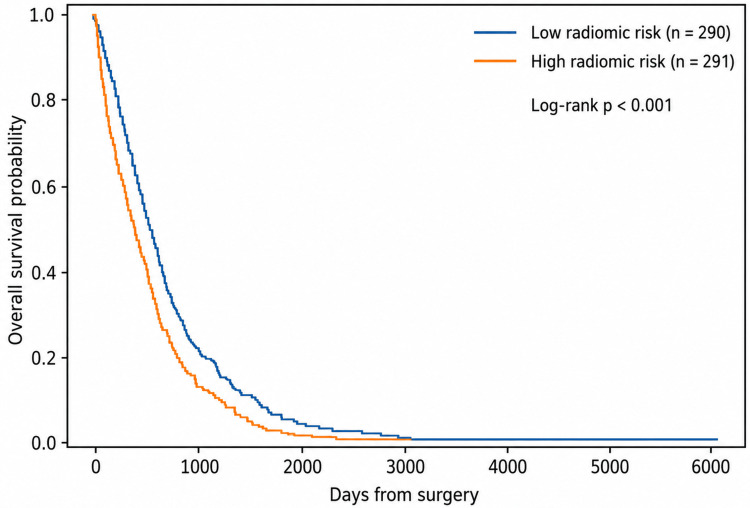
KM curve for UPenn-GBM stratified by locked radiomic risk group High radiomic risk was associated with worse OS (log-rank p < 0.001). KM: Kaplan-Meier, UPenn-GBM: University of Pennsylvania glioblastoma, OS: overall survival

The locked MRI radiomic score demonstrated reproducible prognostic value in the UPenn-GBM external cohort (Table 4; Figure 4). In univariable Cox regression, a higher radiomic score was associated with worse OS (HR 1.634; 95% CI, 1.302-2.050; p = 2.2 x 10^-5^; C-index 0.565). KM analysis showed worse survival in the high radiomic risk group compared with the low radiomic risk group (log-rank p < 0.001; Figure 4). In the primary multivariable external validation model adjusted for age, IDH status, and extent of resection, the radiomic score remained independently associated with OS (adjusted HR 1.354; 95% CI, 1.037-1.769; p = 0.026). The addition of the radiomic score to a clinical model modestly improved discrimination (C-index from 0.626 to 0.632) and model fit (partial AIC from 4756.3 to 4753.3).

**Table 4:** Prognostic performance of radiomics and conventional tumor volume in the UPenn-GBM cohort Univariable, multivariable, and head-to-head Cox proportional hazards analyses evaluating the prognostic performance of the radiomic score and automated tumor volume in the external UPenn-GBM validation cohort. HRs, 95% CIs, and p-values are reported. Multivariable models were adjusted for age, IDH status, and MGMT promoter methylation. C-index (concordance index) is a measure of model discrimination, ranging from 0.5 (no discrimination) to 1.0 (perfect discrimination); C-index values represent the discrimination of the full model, including all covariates. “Same model” in the last column indicates that the C-index reported in the preceding row applies to the joint model that includes both the radiomic score and automated tumor volume as simultaneous predictors. UPenn-GBM: University of Pennsylvania glioblastoma, HRs: hazard ratios, CIs: confidence intervals, IDH: isocitrate dehydrogenase, MGMT: O6-methylguanine-DNA methyltransferase

Model	Covariate	HR (95% CI)	p-value	C-index/model fit
UPenn univariable radiomics	Radiomic score	1.634 (1.302-2.050)	2.2 x 10^-5^	C-index 0.565
UPenn clinical-adjusted radiomics	Radiomic score	1.354 (1.037-1.769)	0.026	Clinical + radiomics C-index 0.632
UPenn head-to-head	Radiomic score	1.626 (1.295-2.042)	<0.001	C-index 0.566
UPenn head-to-head	Automated volume	0.996 (0.981-1.012)	0.655	Same model
UPenn adjusted head-to-head	Radiomic score	1.580 (1.246-2.005)	<0.001	C-index 0.653
UPenn adjusted head-to-head	Automated volume	0.998 (0.982-1.014)	0.806	Same model

Head-to-head comparison favored radiomics over volume

In a direct UPenn head-to-head analysis, radiomics retained prognostic significance, whereas automated tumor volume did not (Table 4; Figure 4). In the unadjusted two-biomarker model, radiomic score was significantly associated with OS (HR 1.626; 95% CI, 1.295-2.042; p < 0.001), whereas automated tumor volume was not (HR 0.996; 95% CI, 0.981-1.012; p = 0.655). In the adjusted head-to-head model including age at scan, IDH status, and MGMT methylation status, radiomic score remained independently prognostic (HR 1.580; 95% CI, 1.246-2.005; p < 0.001), while automated tumor volume remained nonsignificant (HR 0.998; 95% CI, 0.982-1.014; p = 0.806). The adjusted model C-index was 0.653. These findings directly support the conclusion that radiomic phenotyping captured prognostic information not contained in conventional automated tumor volume.

Bootstrap and segmentation sensitivity analyses supported radiomic robustness

**Figure 5:**
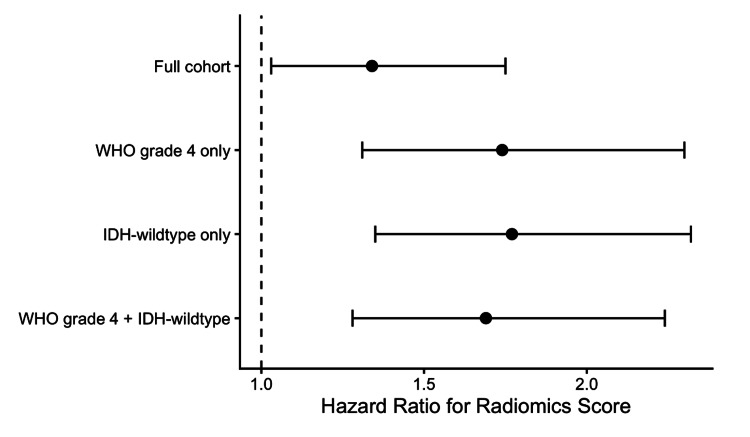
Subgroup forest plot of the radiomic score in UPenn-GBM HRs and 95% CIs were estimated using Cox proportional hazards regression for each subgroup. Radiomic prognostic association remained directionally consistent across the full cohort, WHO grade 4 only, IDH-wildtype only, and WHO grade 4 plus IDH-wildtype subsets. UPenn-GBM: University of Pennsylvania glioblastoma, HRs: hazard ratios, CIs: confidence intervals, WHO: World Health Organization, IDH: isocitrate dehydrogenase

Bootstrap resampling supported stability of the radiomic survival association (Table 5). Subgroup consistency of the radiomic score across clinically relevant patient subsets is illustrated in Figure 5. Over 1,000 successful bootstrap iterations, the median univariable radiomic HR was 1.652, with a 95% bootstrap interval of 1.283-2.098. In the adjusted bootstrap analysis, the median radiomic HR was 1.371, with a 95% bootstrap interval of 1.050-1.838. Although discrimination gains were modest, model fit improved in the bootstrap comparison, with median delta partial AIC of -3.24 favoring the clinical plus radiomic model. Manual-segmentation sensitivity analysis in 145 patients was directionally consistent but underpowered (HR 1.319; 95% CI, 0.832-2.089; p = 0.238; manual KM log-rank p = 0.054). Among 145 overlapping patients, automated and manual radiomic scores were strongly correlated (Pearson r = 0.841, p = 5.39 × 10^-40^; Spearman rho = 0.827, p = 1.53 × 10^-37^), supporting consistency across segmentation approaches.

**Table 5:** Bootstrap and segmentation sensitivity analyses for UPenn radiomic validation Bootstrap and segmentation sensitivity analyses demonstrating stability of the radiomic survival association and consistency of radiomic scores across automated and manual segmentations. UPen: University of Pennsylvania, HR: hazard ratio, AIC: Akaike information criterion, KM: Kaplan-Meier, CI: confidence interval

Analysis	Result
Bootstrap iterations	1,000 successful iterations
Univariable radiomic HR bootstrap median	1.652 (95% interval 1.283-2.098)
Adjusted radiomic HR bootstrap median	1.371 (95% interval 1.050-1.838)
Delta partial AIC bootstrap median	-3.24 (clinical + radiomics favored)
Manual segmentation sensitivity	HR 1.319 (95% CI 0.832-2.089), p = 0.238
Manual KM log-rank	p = 0.054
Automated versus manual score correlation	Pearson r = 0.841; Spearman rho = 0.827

## Discussion

This multi-cohort study compared conventional tumor volume with MRI radiomics for glioma prognostic stratification. The central finding is that tumor volume was prognostic in a post-treatment discovery cohort but did not demonstrate consistent independent prognostic performance across external cohorts. In contrast, a locked MRI radiomic score retained reproducible prognostic value in UPenn-GBM and remained significant when directly modeled alongside automated tumor volume. These results support the concept that radiomic phenotyping captures information beyond simple tumor burden, consistent with prior work showing that medical images can be mined as quantitative data rather than interpreted only visually [4-8].

The MU discovery results show that post-treatment active tumor volume can be clinically meaningful. Active tumor volume remained independently associated with PFS after adjustment for age, grade, IDH status, and MGMT promoter methylation. This finding is intuitive: residual or recurrent active tumor burden after treatment likely reflects biologically aggressive disease and incomplete disease control. However, the external analyses demonstrate the limitations of treating volume as a universally robust prognostic marker. UCSD volume analysis was not significant, UCSF volume was significant only in univariable analysis and lost significance after adjustment, and UPenn automated volume was not associated with OS. Differences in disease setting, endpoint (PFS in MU and UCSD versus OS in UCSF and UPenn), segmentation definition, imaging protocol, and cohort composition likely contributed to this variability. These endpoint differences also preclude direct numeric comparison of HR magnitudes across cohorts; the interpretive weight of the cross-cohort comparison rests on the pattern of significance rather than the consistency of effect sizes.

Radiomic analysis demonstrated a different pattern. In UPenn-GBM, the locked radiomic score was associated with OS, separated KM survival curves, remained significant after clinical adjustment, and retained significance in the head-to-head model with tumor volume. The head-to-head result is especially important because it directly addresses whether radiomics is simply a proxy for tumor size. If the score merely represented tumor burden, the radiomic effect would be expected to weaken substantially when volume was included. Instead, radiomics remained significant while volume remained nonsignificant. This supports the interpretation that the score captures additional phenotype dimensions such as shape irregularity, sphericity, boundary complexity, and intratumoral texture heterogeneity, which are core targets of radiomic feature extraction [4-6,9,17].

The magnitude of performance improvement should be interpreted carefully. Although the radiomic score was statistically robust, the incremental improvement in discrimination over clinical variables alone was modest. Time-dependent receiver operating characteristic analysis over a one-year horizon further illustrated the incremental gain achieved by adding radiomic information to the clinical model (Figure 6). This finding argues against presenting the model as a stand-alone clinical prediction tool. A more appropriate interpretation is that radiomics provides reproducible adjunct prognostic information. This is consistent with how imaging biomarkers may be most clinically useful: not as replacements for age, molecular markers, and treatment variables, but as complementary descriptors of tumor phenotype [2,10,11].

Segmentation sensitivity testing further informs reproducibility. The manual-segmentation subset (n = 145) was underpowered for independent Cox regression validation, given the observed effect size of approximately HR 1.3; roughly 350-400 patients with events would be needed to achieve 80% power, and accordingly, the analysis did not reach statistical significance (HR 1.319, p = 0.238). However, the direction and magnitude of the effect were consistent with the automated segmentation finding, and KM separation was borderline significant (p = 0.054). Critically, automated- and manual-derived radiomic scores were strongly correlated among overlapping patients (Pearson r = 0.841; Spearman rho = 0.827), confirming that the locked score captures a reproducible imaging phenotype across segmentation approaches rather than an artifact of one particular pipeline. This segmentation concordance is an important positive finding for clinical translation, where segmentation variability remains a recognized barrier [9,17].

This study also highlights an important methodological point for imaging biomarker research. Negative or inconsistent performance of a simple comparator biomarker can be informative. Demonstrating that radiomics adds information beyond volume strengthens the biological plausibility and practical relevance of the radiomic model. Without a conventional comparator, an externally validated radiomic score may still be criticized as another statistical signature. With direct comparison, the analysis addresses a more clinically meaningful question: whether high-dimensional imaging phenotyping offers information beyond the measurements radiologists and oncologists already understand [4-6,10,11].

From a clinical imaging perspective, the present findings suggest several potential applications for a validated radiomic risk score in glioma care. At the preoperative stage, a score derived from standard-of-care MRI could provide prognostic stratification prior to surgery and molecular profiling, potentially informing patient counseling and surgical planning. At the post-surgical or pre-adjuvant stage, the score could complement MGMT and IDH status to refine risk group assignment for clinical trial enrollment. For longitudinal surveillance, automated radiomic scoring of follow-up MRI could flag phenotypic changes that precede formal radiographic progression, thereby warranting earlier assessment. Realizing any of these applications will require prospective validation, standardization of feature-extraction pipelines across scanner platforms and acquisition protocols, and integration with clinical decision-support frameworks. The present study does not yet support routine clinical deployment; rather, it identifies a reproducible prognostic signal and establishes a methodological foundation for prospective evaluation. For the practicing radiologist, the key message is that quantitative imaging features extracted from routine clinical MRI sequences encode survival-relevant information that goes beyond what conventional volume measurement captures.

Limitations

This study has several limitations. First, the discovery cohort evaluated PFS, whereas the external validation cohorts evaluated OS, preventing direct comparison of effect sizes across cohorts. Differences in endpoint definition, disease setting (post-treatment versus preoperative), and cohort composition likely contribute to observed variability in volume performance. Nevertheless, the consistent prognostic direction of the radiomic score across independent datasets supports the robustness of the findings. Second, the UPenn-GBM validation cohort as used in this analysis had a 100% event rate, reflecting an ascertainment-censored dataset structure rather than a prospective or consecutive patient sample. While this configuration is inherent to the publicly available data resource, it may bias survival estimates and limit generalizability. The radiomic associations observed in UPenn-GBM should be interpreted as internally consistent prognostic signals rather than definitive clinical effect sizes. Third, the UPenn validation cohort consisted predominantly of high-grade gliomas and contained a limited number of IDH-mutant tumors, which may reduce the ability to assess performance within specific molecular subgroups. Finally, this study was retrospective and used publicly available datasets not originally collected for radiomic biomarker development. Prospective, multi-institutional validation in consecutively enrolled patients will be necessary to determine whether radiomic risk stratification can meaningfully improve clinical decision-making and patient management. This study was reported in accordance with Transparent Reporting of a Multivariable Prediction Model for Individual Prognosis or Diagnosis + Artificial Intelligence (TRIPOD+AI) guidelines [11].

## Conclusions

Across independent glioma cohorts, conventional tumor volume showed inconsistent prognostic performance and failed to remain significant in UPenn head-to-head modeling. In contrast, MRI radiomics remained independently associated with OS and retained significance after adjustment for clinical variables and automated tumor volume. These findings suggest that radiomic phenotyping provides prognostic information beyond simple tumor burden and may offer a more reproducible quantitative imaging framework for glioma risk stratification.
